# Response to a trial on reversal of Death by Neurologic Criteria

**DOI:** 10.1186/s13054-016-1561-5

**Published:** 2016-11-22

**Authors:** Ariane Lewis, Arthur Caplan

**Affiliations:** 1 Division of Neurocritical Care, Departments of Neurology and Neurosurgery, NYU Langone Medical Center, 530 First Avenue, HCC-5A, New York, NY 10016 USA; 2 Division of Medical Ethics, Department of Population Health, NYU Langone Medical Center, 227 East 30th Street 7th Floor, New York, NY 10016 USA

**Keywords:** Brain death, Death by neurologic criteria

In April 2016, plans were announced for a dubious study to examine whether the use of intrathecal bioactive peptides, stem cells, lasers, and median nerve stimulation can effectively reverse death by neurologic criteria (DNC) [1]. The possibility that DNC could be reversed was reported in hundreds of articles around the world [2].

Unfortunately, this study has no scientific foundation [3]. Biomedical science is based upon a quest for knowledge through observation and experimentation. Bioquark’s study allegedly seeks to facilitate this quest. However, scientific inquiry cannot be haphazard, and human studies must be evidence-based and adhere to standards and regulations.

This project was approved internally at a hospital in Rudrapur, India, but was not reviewed by the Indian Council for Medical Research or any external scientific or ethical committees [3]. Although the study is being conducted in India and is not subject to American regulatory governance, Bioquark is based in the United States and the trial is listed on the National Institute of Health’s website, ClinicalTrials.gov. [1] Given the complete absence of foundation for this study and it’s at best, ethically questionable, and at worst, outright unethical nature, this trial would never be approved in the United States [3]. Moreover, Bioquark, who aims to “alter the regulatory state of human tissues and organs,” has an blatant conflict of interest in undertaking this activity [4].

By definition, DNC requires irreversible cessation of all functions of the entire brain, including the brainstem. As such, the proposal that DNC could be reversible is self-contradictory. DNC is well-acknowledged by the medical, legal, and ethical communities to constitute legal death. The public, however, has a poor understanding of DNC [5]. The suggestion that DNC could be reversed provides families of brain dead patients a cruel, false hope for recovery. This is especially so for families that believe in reincarnation [3, 5].

Public understanding of DNC impacts organ and tissue donation, legislation, public policy and clinical scenarios. Knowledge about DNC is often based on stories in the media, but articles about DNC frequently focus on dramatics and fail to educate the public [5].

Because this trial borders on quackery yet has been well-publicized [2], it is the responsibility of the academic community to facilitate a public dialogue about its scientific and ethical shortcomings. Dead means dead. Proposing that DNC may not be final openly challenges the medical-legal definition of death, creates room for the exploitation of grieving family and friends and falsely suggests science where none exists [3].

## Abbreviations

DNC: death by neurologic criteria
